# Acute and sub-acute toxicity of *Echinops kebericho* decoction in rats

**DOI:** 10.1186/s12906-019-2794-z

**Published:** 2020-01-13

**Authors:** Serawit Deyno, Abiy Abebe, Mesfin Asefa Tola, Ariya Hymete, Joel Bazira, Eyasu Makonnen, Paul E. Alele

**Affiliations:** 1 Department of Pharmacology, Mbarara University of Science and Technology, P.O.BOX 1410, Mbarara, Uganda; 2 Department of Pharmacology, School of Medicine, College of Medicine and Health Sciences, Hawassa University, P.O.BOX 1560, Hawassa, Ethiopia; 3 Pharmbiotechnology and Traditional Medicine Center of Excellence (PHARMBIOTRAC), Mbarara University of Science and Technology, P.O. Box 1410, Mbarara, Uganda; 4 Traditional and Modern Drug Research Directorate, Ethiopian Public Health Institute, Addis Ababa, Ethiopia; 5 Department of Pathology, Saint Paul’s Hospital Millennium Medical College, Addis Ababa, Ethiopia; 6 Department of Pharmaceutical chemistry and Pharmacognosy, Addis Ababa University, Addis Ababa, Ethiopia; 7 Department of Microbiology, Mbarara University of Science and Technology, Mbarara, Uganda; 8 Center for Innovative Drug Development and Therapeutic Trials for Africa, College of Health Sciences, Addis Ababa University, Addis Ababa, Ethiopia; 9 Department of Pharmacology and Clinical Pharmacy, College of Health Sciences, Addis Ababa University, Addis Ababa, Ethiopia

**Keywords:** Herbal medicine, Traditional medicine, Safety, plant medicine, Adverse effect

## Abstract

**Background:**

*Echinops kebericho* is widely used for treatment of a variety of diseases including infectious, non-infectious disease and fumigation during child birth. Antibacterial, antimalarial, anti-leshimania, anti-diarrheal and insect repellent activities have been elucidated. Its toxicity profile is not yet investigated and thus this study was to investigate acute and sub-acute toxicity of *E. kebericho* decoctions.

**Methods:**

Acute toxicity study was performed in female Wistar albino rats with single oral dose and followed up to 14 days. The sub-acute oral dose toxicity studies were conducted in rats of both sexes in accordance with the repeated dose 28-day oral toxicity study in rodent OECD guidelines. Physical observations were made regularly during the study period while body weight was measured weekly. Organ weight, histopathology, clinical chemistry and hematology data were collected on the 29th day. Results were presented as mean ± standard deviation. One-way analysis of variance (ANOVA) was performed if assumptions were met; otherwise Kruskal-Wallis analysis was performed.

**Result:**

Oral administration of *E. kebericho* decoction showed no treatment-related mortality in female rats up to the dose of 5000 mg/kg. In sub-acute toxicity studies, no significant treatment-related abnormalities were observed compared to negative controls. Food consumption, body weight, organ weight, hematology, clinical chemistry, and histopathology did not show significant variation between controls and treatment groups. However, creatinine, relative lung weight, triglycerides, and monocytes were lower in treated compared to control groups. Significant variations between male and female groups in food consumption, relative organ weight, hematology, clinical chemistry were observed. Histolo-pathology of high-dose treated groups showed fatty liver.

**Conclusion:**

*Echinops kebericho* showed LD_50_ of greater than 5000 mg/kg in acute toxicity study and is well tolerated up to the dose of 600 mg/kg body weight in sub-acute toxicity study.

## Background

Medicinal plants have played significant roles in the treatment of diseases traditionally and in the development of modern drugs [1]. The use of herbal medicines and its supplements is globally increasing tremendously [2] and about 80% of the world population rely on it for some part of their primary healthcare [3]. Tremendous attention is being given to herbal medicines due to limitation of modern medicine. Dissatisfaction with modern medicine, positive aspects associated with herbal medicine, family traditions were the most commonly mentioned reasons why herbal medicine was preferred [2]. Antimicrobial resistance, increased cost, and safety concerns with modern medicines augmented the interest in traditional medicine. The current health care appears to be shifting to a concoction of numerous phytochemicals with polypharmacological effects acting on multiple pharmacological targets resulting in different biological mechanisms and treating varieties of diseases [4].

Ethiopia has a long tradition of herbal medicine use on which most of the populations rely partly for their primary health care [5]. *Echinops kebericho* Mesfin *(*Family: Asteraceae) is among the many commonly used endemic medicinal plants in Ethiopia [6]. *Echinops kebericho* is an erect perennial herb or bush, commonly forming a massive root stock with grassy stems [7–9]. *Echinops kebericho* contains flavonoids, alkaloids, triterpenoids, resines, saponins and steroids [7, 10]. Dehydrocostus lactone is isolated and identified from the tuber [7, 11, 12]. Saponfiable and unsaponfiable constituents of the fixed oils from this plant were also investigated [13]. Various in vitro and in vivo studies have confirmed the ethnopharmacological claims [10, 11, 14, 15].

Teklehaymanot and his colleagues documented *E. kebericho*‘s diverse ethnopharmacological uses [16]. Its smoke is inhaled for fever, typhoid, tonsilitis, tooth ache, typhus, common cold, cancer, hypertension, colic, cough, and for “evil eye” [17]. In general, it is used in wound infections, lung tuberculosis, leprosy, syphilis, malaria, schistosomiasis, amoebic dysentery, tonsillitis, typhoid fever, cancer, stomach ache, headache, toothache, heart disease, migraine, mental illness, kidney disease, snake repellent, and fumigation during child birth [9, 18, 19]. It is also used in veterinary practice where root tubers are powdered mixed with water and given to cattle and sheep for black leg, respiratory manifestations, liver disease and skin infections [20]. *E. kebericho* is formulated as a water decoction, infusion, smoke inhalant, or chewed [16–19].

Antibacterial activities from crude extracts [21–23] and EOs were reported to be significant [24]. Ivan reported anti-mycobacterium activity of dehydrocostus lactone, isolated from *E. kebericho,* against *Mycobacterium smegmatis* and significant antimicrobial resistance modulatory effects of the isolate were observed from dichloromethane fraction which was elucidated to be (2-(penta-1,3-diynyl)-5-(4-hydroxybut-1-ynyl)-thiophene) [12]. Significant activities were reported against fungi (*Aspergillus flavus* and *Candida albicans*) [23]*,* malaria parasite (*Plasmodium berghe*i) [10], leishmania [11], earthworm [25], and trypanosoma [26]. *Echinops kebericho* extract produced a dose dependent decrease in intestinal propulsion and mean defecation comparable to loperamide [14] and its essential oil produced dose-dependent mosquito repellent activity [27] and moderate larvicidal activity [28].

*Echinops kebericho* is also used in Israel for traditional medicinal purpose [29] where the indigenous knowledge were possibly transferred by Ethiopian Jewish community who went there. It is a source of income for a large number of local people and traditional healers in Ethiopia [17, 23]. Many medicinal plants including *E.kebericho* are blindly considered as safe though they are also well known for toxicity [30]. Drugs coming from medicinal plant like *Atropa belladonna* and *Digitals purpurea* are are appreciably toxic [31, 32]. Investigation of safety profile of the plant is highly required given the pervasive use. With full toxicity profile, its further development and optimal use can be promoted. Herein, we evaluated the acute and sub-acute toxicity of *E. kebericho* decoction in rats to predict its safety in human use, ensure the public safety and promote further development. Rats are recommended lower level of animals for toxicity studies to extrapolate to human biology according to Organization for Economic Cooperation and Development (OECD) safety study guidelines [33, 34]. The finding of the study could also help to guide optimization and validation of the traditional use of the plant.

## Methods

### Plant material collection, extraction and formulation

Fresh plant tuber was collected from Andracha woreda, Sheka zone, South nation nationalities and people region in Ethiopia. The plant was collected after flowering time in November in the morning. This time was selected as this plant was commonly harvested during this season and traditionally herbal medicines are commonly collected during the morning time in the community.. The plant specimen was identified by taxonomist from Addis Ababa University and voucher specimen was deposited at the National Herbarium, Department of Biology, Addis Ababa University with voucher number SD-001/18. The tuber was rinsed with running water to remove dirt materials. In four cycles, five hundred gram of the fresh tuber was pounded and soaked in clean water and then water was added until about 2–3 cm above the plant material. The water was brought to boiling point with strong heat, and then shifted to gentle heat and simmered for another 30–45 min. The mixture was stirred 2–3 times while heating. The supernatant was collected, filtered and evaporated. The filtrate was then lyophilized, wrapped with aluminum foil to prevent photoxidation, and stored in a refrigerator at -20 °C until use. The yield of the extract was 10%, 50 g was obtained from 500 g of pounded tuber. The residue from decoction was dissolved with distilled water, gently stirred, and then administered in calculated doses. Fresh solution of the decoction was made daily to prevent possible spoilage. The maximum volume administered was 2 ml/100 g animal weight in accordance with OECD guideline [34].

### Experimental animals

Wistar albino rats weighing between 250 g – 350 g from Ethiopian Public Health Institute (EPHI) were used. The animals were kept in plastic cages in environmental conditions (22–24 °C, 12 h: 12 h dark/light cycle), fed a standard rodent pellet diet and water ad libitum. Food consumption was measured by subtracting the total amount of food remaining from that of the total served. All experimental rats were acclimatized for two weeks before experiment. In acute toxicity study, rats were housed together in groups of three for groups of control, 300 mg/kg, 2000 mg/kg and 500 mg/kg while each group in sub-acute toxicity study comprised of ten animals, five male and five female. A total of five same sex rats of the same group were housed together. The animals were allocated into experimental groups by simple randomization using lottery method. For the purposes of identification the rats were marked using permanent marker in their tail. The study was conducted with proper animal handling under well-founded conditions in accordance with the recommendations of guide for the care and use of animals [35] and institutional ethical review was obtained to carry out the study. At the end of the experiment the animals were sacrificed using cervical dislocation with all possible effort being given to minimize suffering. The ARRIVE guidelines [36] were used for reporting the study and the checklist is added as Additional file 1.

### Acute toxicity study

Acute toxicity study was performed in female rats in a stepwise procedure with the use of 3 animals per step as recommended in acute oral toxicity class method OECD 423 guideline [33]. The animals were fasted overnight and provided with only water, and then decoction was administered by oral gavage starting with 300 mg/kg and next animal group was dosed up based on the response [33]. Animals were observed closely for the first 4 h, for any toxicity manifestation, like increased motor activity, salivation, convulsion, coma, and death. Subsequent observations were made at regular intervals for 24 h. The animals were kept under further follow-up for 14 days and the number of rats that died within the study period was noted, if any. At the end of the experiment, the animals were sacrificed to examine gross anatomy. Lethal dose in 50 % (LD_50_) was determined in accordance with the guideline principle [33].

### Sub-acute toxicity study

The sub-acute oral dose toxicity studies were conducted in rats of both sexes in accordance with the repeated dose 28-day oral toxicity study in rodents, OECD 407 guideline [34]. Doses of the decoction (150 mg/kg, 300 mg/kg and 600 mg/kg) and water (used as control) were administered by oral gavage once daily for 28 consecutive days. The doses were determined based on the finding of the acute toxicity study and in accordance with ethnopharmacological claims. The control groups received the same volume of vehicle (water) as the test animals. All animals were observed daily for mortality, signs and symptoms of toxicity. Detailed clinical observations were recorded on day 1 (prior to test substance administration) and daily thereafter. These observations were conducted while handling the animal and with the animal placed in good light condition. Signs that were observed included respiratory rate, changes in skin fur, eyes and mucous membranes, occurrence of secretions and excretions, and autonomic activity (e.g., lacrimation, piloerection, pupil size, and unusual respiratory pattern). Behavioral parameters, changes in gait, posture, and response to handling, as well as the presence of clonic or tonic movements, stereotypies (e.g., excessive grooming, repetitive circling), and bizarre behavior (e.g., self-mutilation, walking backwards) were noted. Body weights were measured and recorded twice during acclimatization, at the beginning and then after every week.

At the end of the study, all animals fasted overnight (water ad libitum) and, on 29th day, the animals were weighed and blood collected for hematological and biochemical analysis by cardiac puncture under chloroform anesthesia and then the animals were sacrificed by cervical dislocation. Finally body organs were harvested for detailed gross anatomy, organ weight and histo-pathological investigation. Blood was collected in a pre-calibrated tube containing EDTA for hematology assessments. Hematological autoanalyzer was used to analyze hematological parameters, including red blood cell (RBC) count, white blood cell (WBC) count, hemoglobin (Hb), hematocrit (Hct), mean cell hemoglobin (MCH), mean cell volume (MCV), mean cell hemoglobin concentration (MCHC), platelet count, and others. Blood samples were centrifuged at 3000 rpm for 10 min for biochemical analysis. The serum was carefully aspirated with a micro-pipette into sample bottles for the various biochemical assays. The clinical chemistry parameters were assessed using isolated serum samples analyzed under an automatic analyzer. Analytes included aspartate transaminase (AST), alanine transaminase (ALT), alkaline phosphate (ALP), total protein (TP), total cholesterol (TC), blood urea nitrogen (BUN), creatinine (Crea), total bilirubin (T-Bili), direct bilirubin (Bili-dir), albumin (Alb), triglycerides (TG), and glucose (Glu).

### Organ weight determination and histopathology study

The rats were dissected and the liver, kidneys, spleen, lung, and heart, were excised and absolute weights determined. The relative organ weight, defined as the organ weight per 100 g of body weight at sacrifice was calculated. The specimens for histopathology were fixed in 10% neutral buffered formalin at 18 °C. About 3–4 mm in thickness of each specimen of liver, kidney, and spleen were cut and stained with hematoxylin and eosin stain following standard laboratory procedures. The stained sections were examined under microscope for cellular damage or change in morphology of particular tissue.

### Statistical analysis

GraphPad Prism version 5 statistical software was used for the analysis. Data were presented as mean (Standard deviation, SD). Bartlett’s test for equality of variance and D’Agostino & Pearson omnibus normality test were performed to determine whether assumptions of One-way analysis of variance (ANOVA) were met. If assumptions were met, ANOVA was performed; if not Kruskal-Wallis analysis was performed. Either Tukey or Dunn’s Multiple Comparison Test were performed for significant values in post hoc analysis. The *P* < 0.05 was considered significant.

## Results

Experimental animals were of full health during the experimental allocation and naive to test substance. A total of 6 animals were used in acute toxicity study and followed up to 14 days of treatment. Forty animals were enrolled in sub-acute toxicity studies and 38 animals completed the treatment making 95% completion rate. Two animals died due cases unrelated to treatment during experimental period as confirmed by histopathological findings.

### Effect on acute toxicity

**Fig. 1:**
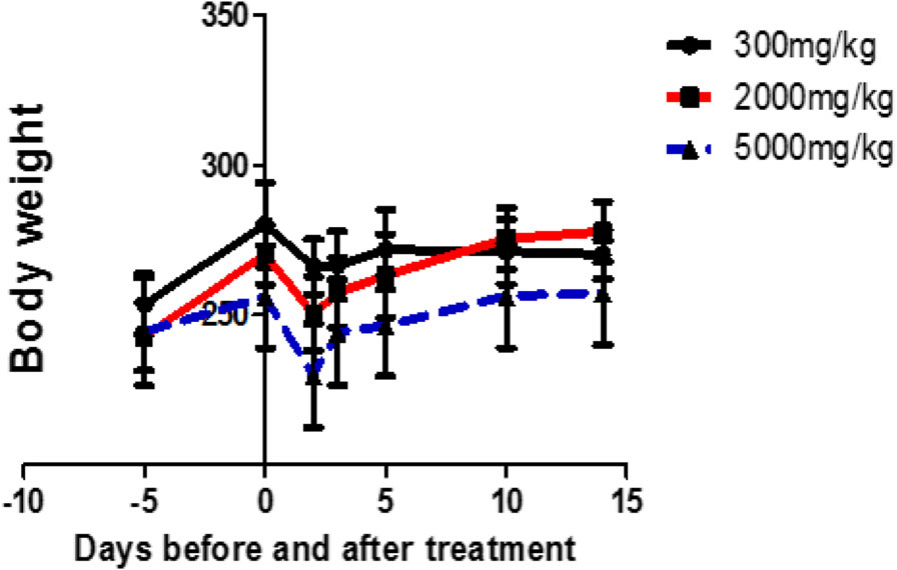
Effect of *E. kebericho* decoction on body weight as function of time (in days) after acute exposure to various dose levels. The body weight appears to decrease just after administration in all of the treatment wings

Oral administration of *E. kebericho* decoction showed no treatment-related mortality up to dose of 5000 mg/kg and no significant treatment related morbidity was observed. However, piloerection, muscle twinge, and lethargy were observed immediately after the treatment with 5000 mg/kg which ceased within 5 h. Effects of decoction on body weight showed significant variations in mean body weight of 300 mg/kg treated group compared to 5000 mg/kg treated, *p*-value = 0.0055. Difference in rank sum of body weight of 300 mg/kg treated group compared to 5000 mg/kg was 9.833. There was a higher drop in body weight of those treated with 5000 mg/kg during the first day of treatment compared to either 300 mg/kg or 2000 mg/kg. Though statistically non-significant, the variations between those treated with 300 mg/kg and 2000 mg/kg or 2000 mg/kg and 5000 mg/kg appears to be treatment related in which higher dose treated groups showed a drop in body weight as shown in Fig. 1.

### Effect on sub-acute toxicity

#### Effect on body weight, food consumption and relative organ weight

**Fig. 2:**
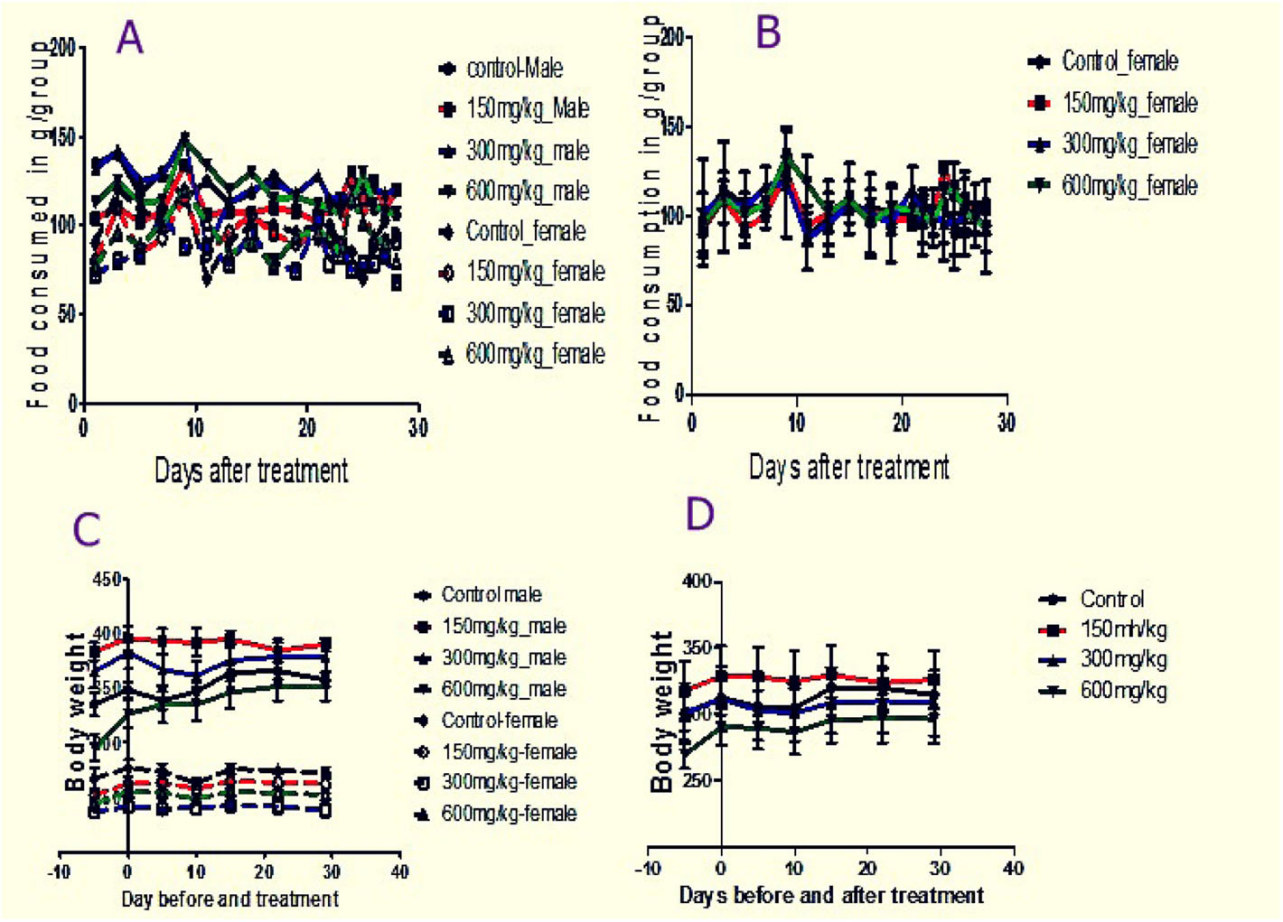
Food consumption pattern as a function of time (in days) among different experimental groups; males and females separately analyzed (**a**) and males and females of the same group combined together, (**b**), Body weight as a function of time (in days) among different experimental groups, males and females analyzed separately (**c**) and males and females combined together (**d**)

Comparison of food consumption and body weight did not show statistically significant variations among treated and control groups of rats, *p*-value =0.6720 and *p*-value = 0.1353 respectively. However, comparison of male and female groups showed significant variation in food consumption, males consumed more compared to females; *p*-value < 0.0001. Although both male and female of nearly the same age were enrolled in the treatment, baseline weights of males were greater compared to females, Fig. 2c. Figure 2c demonstrates that females’ body weight remains constant throughout the treatment while males’ increases and then falls. However, Fig. 2d showed combined groups of rat body weight picking from 10 to 14 days after treatment then falling down.

Linear regression revealed weight gain among control and highest dose treated male groups whereas all other groups did not show changes in body weights as the regression slopes were not significantly different from zero. When males and females weights were combined together, no significant changes in weight as a function of time (in days) were observed (Table 1). Linear regression also showed a decrease in food consumption rate as function of time (in days) in control males and combined controls. No significant changes in consumption as function of time (in days) were observed among other groups (Table 1).

**Table 1:** Linear regression of body weight in experimental rats

Group	Weight gain				Food consumption			
	Slope (95%CI)	*p*-value	R^2^	Interpretation	Slop (95%CI)	*p*-value	R^2^	Interpretation
Control-Female	0.08454 ± 0.1586	0.6132	0.04522	No change	− 0.5674 ± 0.3562	0.1307	0.1369	No change
150 mg/kg-Female	0.1919 ± 0.1185	0.1566	0.3040	No change	0.1455 ± 0.3601	0.6915	0.01010	No change
300 mg/kg-Female	0.1305 ± 0.1144	0.2973	0.1784	No change	−0.2414 ± 0.2905	0.4183	0.04135	No change
600 mg/kg-Female	0.1459 ± 0.1225	0.2784	0.1914	No change	−0.01497 ± 0.3343	0.9648	0.0001253	No change
Control-Male	0.7122 ± 0.2279	0.0205	0.6194	Gain	−0.8286 ± 0.1760	0.0002	0.5808	Decreased
150 mg/kg-Male	−0.3867 ± 0.2712	0.2037	0.2532	No change	0.3500 ± 0.2481	0.1776	0.1106	No change
300 mg/kg-Male	0.3133 ± 0.2192	0.2028	0.2540	No change	−0.5587 ± 0.3436	0.1235	0.1418	No change
600 mg/kg-Male	1.410 ± 0.3622	0.0080	0.7164	Gain	−0.5029 ± 0.2937	0.1062	0.1548	No change
Combined male and female								
Control	0.3984 ± 0.9936	0.6945	0.01135	No change	−0.6980 ± 0.2965	0.0245	0.1402	Decreased
150 mg/kg	−0.09742 ± 1.511	0.9495	0.0002968	No change	0.2477 ± 0.2676	0.3610	0.02459	No change
300 mg/kg	0.2219 ± 1.632	0.8938	0.001319	No change	−0.4000 ± 0.4451	0.4392	0.02320	No change
600 mg/kg	0.7781 ± 1.073	0.4802	0.03621	No change	−0.2589 ± 0.3308	0.4392	0.01770	No change

No significant differences occurred in relative organ weights (liver, kidney, heart, spleen) of treated and control groups (Table 2). However, there were significant differences in mean relative lung weight of 600 mg/kg treated group compared to the control ones. Relative organ weights of were higher in males compared to females according to post-hoc analysis. Mere observation of absolute organ weights showed variations but this doesn’t hold true with relative organ weight. All evaluated internal organs (right kidney, left kidney, spleen, lung, liver, and heart) showed significant variations among males and females.

**Table 2:** Effects of 28 days oral administration of *E. kebericho* decoction on absolute organ weight and relative organ weight

Organs	Male				Female				*P*-value
	control	150 mg/kg	300 mg/kg	600 mg/kg	Control	150 mg/kg	300 mg/kg	600 mg/kg	
Absolute weight									
Liver (in g)	11.49 ± 1.04	12.13 ± 1.21	11.89 ± 2.41	9.19 ± 0.96	9.85 ± 2.18	9.42 ± 0.89	7.13 ± 0.56	9.12 ± 0.72	–
Right kidney (in g)	1.114 ± 0.17	1.22 ± 0.17	1.072 ± 0.17	1.04 ± 0.12	0.91 ± 0.12	0.94 ± 0.10	0.77 ± 0.08	0.86 ± 0.07	–
Left kidney (in g)	1.16 ± 0.16	1.25 ± 0.22	1.02 ± 0.13	0.98 ± 0.08	0.89 ± 0.08	0.912 ± 0.14	0.79 ± 0.09	0.87 ± 0.06	–
Spleen (in g)	0.53 ± 0.04	0.66 ± 0.13	0.61 ± 0.16	0.65 ± 0.11	0.59 ± 0.16	0.63 ± 0.08	0.67 ± 0.07	0.63 ± 0.10	–
Lung (in g)	1.80 ± 0.30	1.86 ± 0.14	1.59 ± 0.13	1.59 ± 0.18	1.65 ± 0.13	1.44 ± 0.06	1.30 ± 0.13	1.19 ± 0.20	–
Heart (in g)	1.35 ± 0.042	1.42 ± 0.23	1.33 ± 0.20	1.19 ± 0.11	1.04 ± 0.04	1.02 ± 0.07	1.02 ± 0.13	1.02 ± 0.06	–
Relative organ weight (Organ weight in gram/100 g of animal weight)									
Liver	3.21 ± 0.26	3.12 ± 0.21	3.13 ± 0.48	2.62 ± 0.35	3.61 ± 0.70	3.60 ± 0.18	3.00 ± 0.24	3.63 ± 0.37	0.0045*/0.2187
Right kidney	0.31 ± 0.04	0.31 ± 0.03	0.28 ± 0.03	0.29 ± 0.02	0.33 ± 0.04	0.36 ± 0.03	0.32 ± 0.03	0.34 ± 0.03	0.0497*/0.2743
Left kidney	0.32 ± 0.04	0.32 ± 0.05	0.27 ± 0.03	0.28 ± 0.02	0.33 ± 0.04	0.35 ± 0.03	0.33 ± 0.03	0.34 ± 0.02	0.0206*/0.2759
Spleen	0.15 ± 0.02	0.17 ± 0.03	0.16 ± 0.03	0.18 ± 0.04	0.22 ± 0.05	0.24 ± 0.04	0.28 ± 0.03	0.25 ± 0.03	0.0013* /0.3968
Lung	0.50 ± 0.06	0.48 ± 0.04	0.42 ± 0.02	0.45 ± 0.04	0.61 ± 0.05	0.55 ± 0.05	0.54 ± 0.04	0.47 ± 0.08	0.0015* /0.0277**
Heart	0.38 ± 0.02	0.36 ± 0.05	0.35 ± 0.04	0.34 ± 0.02	0.38 ± 0.01	0.39 ± 0.02	0.43 ± 0.07	0.40 ± 0.04	0.0409*/0.9093

* Significant *p*-value when males and females are treated separately, **Significant *p*-value when males and females are grouped together

#### Effect on clinical chemistry and hematology

Except for monocytes, all hematological parameters did not show significant variations among the different groups, Table 3. Significant variations in mean differences between female and male groups were observed in RBC, MCHC, RDW-SD, RDW-CV, and monocytes where males showed higher value compared to females, while a higher MCV values was observed in females.

**Table 3:** Mean hematological parameters for 28 week repeated oral doses of *E. kebericho *decoction treated rats

Parameter	Male				Female				*P*-value
	Control	150 mg/kg	300 mg/kg	600 mg/kg	control	150 mg/kg	300 mg/kg	600 mg/kg	
WBC × 10^3^ /μL	6.73 ± 4.20	7.31 ± 3.92	8.91 ± 1.79	8.34 ± 2.62	6.62 ± 1.16	5.88 ± 3.00	5.41 ± 1.98	6.60 ± 2.94	0.4956/0.8792
RBC ×10^6^ /μL	8.80 ± 0.19	8.70 ± 0.56	8.78 ± 0.42	8.91 ± 0.89	8.26 ± 0.73	7.74 ± 0.38	7.93 ± 0.54	8.13 ± 0.37	0.0194*/0.6137
HGB (g/dl)	16.14 ± 0.37	15.88 ± 0.90	16.08 ± 0.60	16.46 ± 1.56	15.78 ± 1.42	15.26 ± 0.25	15.42 ± 0.96	15.70 ± 0.7280	0.5654/0.6188
HCT (%)	45.74 ± 1.36	44.98 ± 2.53	46.64 ± 1.86	47.64 ± 3.31	45.82 ± 3.54	44.40 ± 0.98	44.06 ± 2.00	44.80 ± 2.65	0.2559/0.5777
MCV (fL)	51.96 ± 1.49	51.68 ± 0.71	53.18 ± 1.73	53.50 ± 2.56	55.56 ± 1.50	56.46 ± 1.12	54.26 ± 1.46	55.06 ± 1.62	0.0023*/0.8887
MCH pg	18.34 ± 0.30	18.25 ± 0.19	18.32 ± 0.32	18.46 ± 0.45	19.10 ± 0.45	19.16 ± 0.19	19.26 ± 0.57	19.30 ± 0.29	0.0004*/0.6543
MCHC (g/dL)	35.28 ± 0.54	35.33 ± 0.13	34.48 ± 0.73	34.48 ± 1.10	34.42 ± 0.49	33.92 ± 0.54	35.44 ± 0.25	35.08 ± 0.63	0.0143*/0.7061
PLT ×10^3^ /μL	1072 ± 90.91	993.0 ± 32.37	1066 ± 89.12	1037 ± 142.9	1083 ± 56.89	1052 ± 72.99	1091 ± 51.52	820.6 ± 372.1	0.4173/0.2238
RDW-SD(%)	28.78 ± 0.81	29.28 ± 0.93	29.82 ± 1.18	30.24 ± 0.30	29.26 ± 0.94	29.32 ± 1.22	27.90 ± 1.00	28.50 ± 1.39	0.0331*/0.7570
RDW-CV (fl)	19.28 ± 0.23	19.40 ± 0.94	19.46 ± 0.69	19.95 ± 0.74	17.96 ± 1.24	17.28 ± 0.70	17.60 ± 1.11	17.60 ± 1.08	0.0012*/0.8976
PDW (%)	8.68 ± 0.22	8.52 ± 0.28	8.24 ± 0.11	8.60 ± 0.26	8.22 ± 0.41	8.38 ± 0.28	8.12 ± 0.22	8.28 ± 0.33	0.0429*/0.1478
MPV ×10^3^ /μL	7.78 ± 0.16	7.80 ± 0.14	7.64 ± 0.15	7.80 ± 0.27	7.58 ± 0.22	7.74 ± 0.09	7.60 ± 0.16	7.72 ± 0.23	0.3858/0.2674
P-LCR (%)	9.64 ± 1.01	9.72 ± 1.16	8.66 ± 0.62	9.70 ± 1.70	8.26 ± 1.36	9.20 ± 0.69	8.34 ± 1.18	9.12 ± 1.18	0.3365/0.2621
PCT (%)	0.83 ± 0.06	0.78 ± 0.02	0.81 ± 0.06	0.81 ± 0.09	0.82 ± 0.04	0.81 ± 0.06	0.83 ± 0.03	0.63 ± 0.28	0.5053/0.2593
NEUT (%)	16.54 ± 1.43	22.70 ± 7.31	22.03 ± 6.05	25.66 ± 7.47	17.50 ± 3.37	16.64 ± 3.75	21.90 ± 7.15	17.98 ± 6.54	0.1442/0.3017
LYMPH (%)	77.18 ± 1.63	71.18 ± 7.63	71.58 ± 6.20	67.86 ± 7.63	75.84 ± 4.44	74.36 ± 4.63	74.12 ± 7.43	75.58 ± 6.328	0.4319/0.3016
MONO (%)	5.14 ± 1.43	4.72 ± 1.79	4.65 ± 2.05	5.56 ± 0.69	5.12 ± 1.43	7.52 ± 1.61	2.46 ± 1.42	3.22 ± 2.87	0.0231*/0.0295**
EO (%)	0.86 ± 0.21	1.28 ± 0.44	1.58 ± 0.15	0.78 ± 0.11	1.40 ± 0.55	1.22 ± 0.37	1.24 ± 0.58	2.10 ± 2.08	0.0346*/0.1318
BASO (%)	0.28 ± 0.25	0.12 ± 0.10	0.82 ± 1.44	0.14 ± 0.11	0.14 ± 0.05	0.26 ± 0.32	0.08 ± 0.11	0.52 ± 0.73	0.5610/0.8971
IG (%)	0.16 ± 0.15	0.25 ± 0.17	0.17 ± 0.096	0.24 ± 0.15	0.20 ± 0.19	0.08 ± 0.13	0.24 ± 0.055	0.25 ± 0.21	0.6349/0.6137

*Significant *p*-value when males and females are treated separately, ** significant *p*-value when males and females are grouped together; Where, *PLT* Platelets, *RBC* Red blood cells, *WBC* White blood cells, *MCV* Mean corpuscular volume, *MCH* Mean corpuscular hemoglobin, *MCHC* Mean corpuscular hemoglobin concentration, *RDW* Red cell distribution width, *PDW* Platelet volume distribution width, *HCT* Hematocrit, *HGB* Hemoglobin

Clinical chemistry results did not show significant differences in values between treated groups and control ones, except for creatinine, direct bilirubin and TG levels (Table 4). Significantly lower level of TG was observed in those treated with 300 mg/kg and 600 mg/kg compared to the control group. Significant variations were also observed in the level of direct bilirubin among those treated with 150 mg/kg and those with 150 mg/kg versus 300 mg/kg compared to control group (Table 4). Post-hoc analysis showed cholesterol, TG, creatinine, ALP and albumin levels were lower in females compared to males and higher direct bilirubin females compared to males.

**Table 4:** Mean clinical chemistry parameters for 28 week repeated oral doses of E. kebericho decoction treated rats

Parameter	Male				Female				*P*-value
	control	150 mg/kg	300 mg/kg	600 mg/kg	Control	150 mg/kg	300 mg/kg	600 mg/kg	
AST (U/L)	260.2 ± 30.06	288.3 ± 70.71	241.6 ± 76.59	173.5 ± 21.89	304.2 ± 256.1	190.6 ± 20.74	193.6 ± 29.08	301.6 ± 309.4	0.0559 /0.1173
ALT (U/L)	84.20 ± 7.73	80.75 ± 6.99	88.00 ± 14.37	64.20 ± 6.30	123.0 ± 128.3	70.40 ± 4.88	56.60 ± 7.16	83.80 ± 22.11	0.0036 */0.6072
ALP (U/L)	74.60 ± 20.26	71.25 ± 23.91	106.6 ± 56.20	131.3 ± 16.88	197.8 ± 126.4	52.20 ± 16.98	45.40 ± 46.41	83.20 ± 32.82	0.0162 */0.0577
Creatinine (mg/dl)	0.60 ± 0.07	0.52 ± 0.10	0.52 ± 0.16	0.30 ± 0.12	0.60 ± 0.07	0.52 ± 0.08	0.46 ± 0.05	0.42 ± 0.11	0.0077 */0.0005 **
Urea (mg/dL)	40.30 ± 2.94	42.00 ± 1.26	50.48 ± 6.58	41.50 ± 3.90	47.22 ± 6.14	48.30 ± 7.97	42.72 ± 5.02	43.88 ± 3.51	0.1288/0.5085
Protein total (g/dl)	5.90 ± 0.73	6.30 ± 0.74	6.32 ± 1.00	5.80 ± 0.63	7.06 ± 0.56	6.60 ± 1.40	6.48 ± 0.58	6.62 ± 0.43	0.1531 /0.9694
Albumin (g/dl)	3.20 ± 0.12	3.22 ± 0.17	3.14 ± 0.21	3.05 ± 0.10	3.56 ± 0.24	3.36 ± 0.15	3.38 ± 0.22	3.42 ± 0.228	0.0122*/0.6266
Glucose (mg/dl)	127.4 ± 42.51	178.8 ± 69.63	181.6 ± 64.31	124.8 ± 33.04	116.2 ± 17.71	193.0 ± 45.67	140.0 ± 31.16	188.6 ± 62.42	0.0457*/0.0581
Bil-total (mg/dl)	0.18 ± 0.04	0.38 ± 0.15	0.20 ± 0.0	0.28 ± 0.10	0.26 ± 0.05	0.16 ± 0.05	0.24 ± 0.09	0.34 ± 0.05	0.0036*/0.0644
Bil-direct (mg/dl)	0.10 ± 0.0	0.32 ± 0.10	0.14 ± 0.06	0.12 ± 0.05	0.14 ± 0.06	0.20 ± 0.07	0.14 ± 0.06	0.22 ± 0.11	0.0129*/0.0065**
TG (mg/dl)	79.40 ± 20.19	79.50 ± 8.96	66.60 ± 20.19	51.25 ± 13.45	98.00 ± 41.43	75.20 ± 14.45	58.00 ± 17.56	62.00 ± 9.38	0.0338*/0.0042**
Cholesterol (mg/dl)	33.60 ± 10.85	79.50 ± 16.76	65.60 ± 15.63	38.50 ± 3.70	84.60 ± 37.63	81.80 ± 29.78	81.20 ± 18.10	80.60 ± 12.52	0.0013*/0.2970

*Significant *p*-value when males and females are treated separately, **Significant *p*-value when males and females are grouped together: Where, *ALP* Alkaline phosphatase, *ALT* Alanine aminotransferase, *AST* Aspartate Aminotransferase, *TG* Triglycerides, *Bil-total* Bilirubin Total, *Bil-direct* Direct Bilirubin3.2.3. Post-hoc analysis result

Post-hoc analysis (Tukey or Dunn’s Multiple Comparison Test) results revealed that relative lung weights, creatinine, direct bilirubin, TG and monocytes levels showed significant differences among the treatment groups The relative lung weight was significantly higher among 600mg/kg treated group compared to control (0.09221[95%CI: 0.007704, 0.1767]). The group treated with 150 mg/kg decoction showed a higher monocyte level compared to those treated with 300 mg/kg (2.844 [95%CI: 0.3709, 5.318]). Creatinine level decreased in 300 mg/kg and 600 mg/kg treated groups compared to the control group with rank sum difference respectively of 12.70 and 20.07. Lower level of direct bilirubin was observed in controls compared to 150 mg/kg treated groups (rank sum difference − 15.48). The level of TG was reduced in highest dose treated group compared to controls (rank sum difference = 15.84).

#### Effect on histopathology

**Fig. 3:**
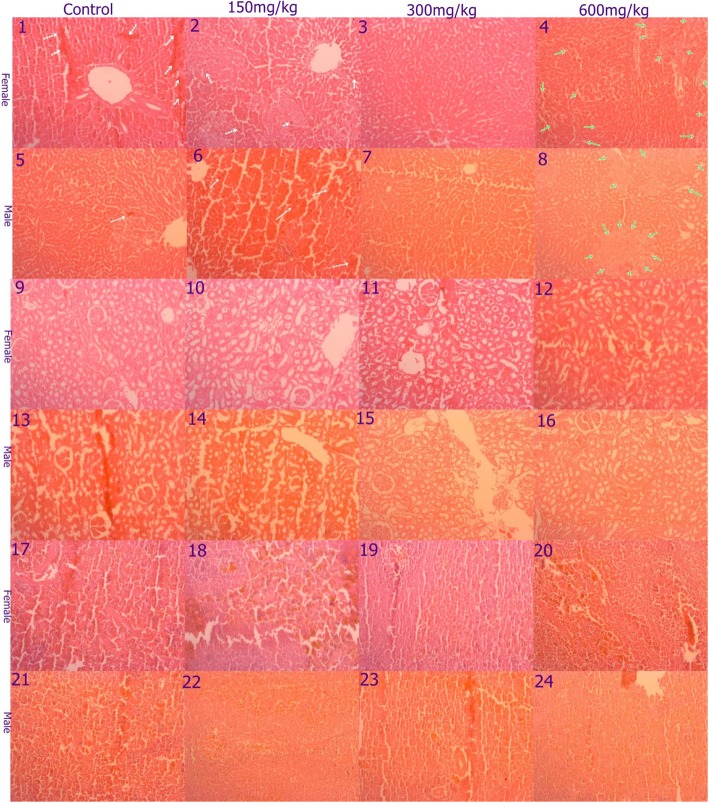
Photomicrograph of liver, kidney and spleen in sub-acute oral toxicity. Mild spotty liver necrosis was observed in either sex of of control and low dose (1, 2, 5, and 6) indicated by white arrow. Groups in medium dose group of either sex showed normal morphology (3 and 7). Liver of high dose treated groups in either sex showed moderate fatty change (green arrow), 4 and 8. Kidney of all groups in box sex showed normal morphology (9–16). Spleen of all groups showed normal but mild sinusoidal congestion (17–24)

Gross anatomical examination of the vital organs (liver, kidney, heart, lung and spleen) in acute and sub-acute oral toxicity study did not reveal any gross pathological lesions. Histo-pathological examinations revealed mild alterations in the liver, however, it appears unrelated to treatment as the pathological changes were also observed in control group. Necrosis was observed in controls, low- and high-dose treated groups, but medium dose treated groups did not show such necrosis. Fatty liver was observed with high dose of treatment. The photomicrographs of the kidney showed normal morphological architecture among the different dose levels and controls. Mild sinusoidal spleen congestion was observed with all treated groups including control group (Fig. 3).

## Discussion

This study presented the first comprehensive investigation on the safety of a commonly used endemic medicinal plant in Ethiopia. It revealed that the LD_50_ of *E. kebericho* decoction could be greater than 5000 mg/kg, putting the plant in GHS Category 5. The 28 day repeated dose toxicity study revealed that *E. kebericho* did not show significant alterations in hematology, clinical chemistry, or derangements in histology up to 600 mg/kg/day compared to controls. Food consumption and body weight were similar in treated groups compared to control. Decrease in food consumption rate was observed across all groups as a function of time (in days). The relative organ weight, hematology and chemistry findings were almost similar in treated compared to controls with little variations in some groups which could be considered as incidental and inconsequential. The level of creatinine, relative lung weight, triglycerides, and monocytes were significantly lower in treated groups compared to controls or higher dose treated groups compared to lower dose treated groups. Food consumption, relative organ weight, hematology, and clinical chemistry showed sex dependent variations.

The findings of the acute toxicity study demonstrated the tolerability of the *E. kebericho* decoction to the maximum dose recommended (5000 mg/kg). However, significant weight loss was observed with 5000 mg/kg treated group signposting sub-lethal toxicity of the extracts. The weight loss could be due to gastrointestinal irritation which reduces food consumption in contrast to metabolic derangement [37]. To protect animal welfare, testing animals in Category 5 (5000 mg/kg) ranges is discouraged [33] without good justification, yet this experiment was conducted with the assumption that there could be a possibility of exposure to higher dose in both human and veterinary medicine as ethnopharmacolgical studies reveal [18, 20].

Food consumption and body weight was almost constant in all combined groups as shown from linear regression results where the slopes were not significantly different in almost all groups. However, there was weight gain in control male rats while there is reduced food consumption. This could be attributed to edema or some unknown reasons. Food consumption could remain constant but failure to gain weight could be due to experimental stress such as use of oral gavage and/or disturbances from handling [38]. Adjustment to handling or study related procedures is common where pretreatment values differs from post-treatment in control animals [39]. Significant difference observed in relative lung weight between highest doses treated group (600 mg/kg) and control could be attributed to incidental events or some factors related to treatment which the current study could not uncover.

Hematological and clinical chemistry parameters are good indicators in determining toxicity [40]. Blood parameter analysis is appropriate to risk evaluation as the hematological system has a higher prognostic value for toxicity [39, 41]. In the present study, almost all hematological parameters did not show significant variation among treated and control groups indicating the possible absence of hematological toxicity. Reduction in monocyte counts could be expected during severe bone marrow toxicity [42], like administration of cytotoxic chemotherapeutic agents [43]. This finding could have been corroborated with other incidents where bone marrow toxicity could bring changes, such as reduction in RBC. But, no reduction in RBC could mean absence in bone marrow toxicity which rules out the reduction in monocyte level as incidental.

Liver and renal function tests reveal hepatic and renal toxicity as target organs due to involvement in elimination of xenobiotics. Drug-induced liver injury is a result of its anatomical proximity to the portal blood supply, its involvement in concentration, bio-transformation and elimination of xenobiotics [44]. Preclinical safety assessments could connote any potential adverse effects that could occur to the end users [45]. Liver function test and histo-pathological findings of current study did not show hepatotoxicty. Yet, mild necrosis was observed in low dose treated and control groups, and moderate fatty liver was observed with higher-dose treated group but those treated with medium dose showed normal liver morphology. As alteration in histological architecture, potentially pathological changes, was observed including in the control but not the medium-dose treated group. The mild necrosis observed in control and low-dose treated groups could be considered as subsidiary and negligible but moderate fatty liver with high-dose treated group could potentially be linked with the treatment. The fatty change observed with high-dose treated group could be due to liver problem or some other unknown changes. Problems in muscles mass could have lowered creatinine level in high-dose treated groups compared to control [46].

Lower level of creatinine was observed in higher dose compared to lower dose treated group or controls in this study. Mean reference serum creatinine levels in the rats aged less than six months is 0.6 mg/dl ± 0.12 and 0.7 mg/dl ± 0.13; and the tenth percentile value is 0.5 mg/dl for both males and females [47]. While the control value exactly matches the reference value given, the high dose treated group showed significantly lower level. This possibly indicated some derangements resulting in lowered creatinine level. Histo-pathological changes observed in kidney and spleen could only be considered as incidental and spontaneous as they were also observed in control group and very mild. The low level of serum creatinine could not be associated with kidney disease and the possible explanation for reduction, but may be associated either to liver function problem or muscle mass reduction.

Sex dependent variations in food consumption where males consumed more compared to females could only be attributed physiological variation between male and female. Similar statistically significant variations were also observed from historical data in control Sprague-Dawley rats in pre-clinical toxicity studies [40] and study by Lillie et al., 1996 [48] revealed that male rats generally have a higher HCT, WBC TG, ALP, ALT, RBC, HGB, PLT, and glucose compared to females. Toxicity studies in animals often deliver valued evidence to predict adverse effects of new drugs in humans. Toxic effects can vary between species but can easily be extrapolated to human. Comparative drug disposition data in corresponding with the pilot toxicity studies in different species can provide a strong basis for appropriate toxicity study designs in human [49]. The current study could therefore provide a stepping stone for possible toxicity study in human.

## Conclusion

*E. kebericho* decoction can be considered Category 5 in accordance with GHS safety category in acute toxicity study and well tolerated up to 600 mg/kg/body weight in sub-acute toxicity study. *E. kebericho* decoction did not cause any significant toxicity resulting in death, or produce any hematological, serum chemical alteration, or histo-pathological derangements. However, significant reductions in the levels of creatinine and TG in high-dose treated groups could be related to mild liver toxicity as fatty liver was observed in liver tissue of high-dose treated group. This study demonstrates the relative tolerability of *E. kebericho* decoction up to 600 mg/kg suggesting its safety and potential candidature as a future safe pharmaceutical product. Given the polypharmacological effect elucidated in previous studies and the tolerability in the current study, there is a need for pharmaceutical product development.

## Supplementary information

**Additional file 1.** The ARRIVE Guidelines Checklist.

## Data Availability

The dataset supporting the conclusions of this article is included within the article.

## Abbreviations

ALP: Alkaline phosphatase
ALT: Alanine aminotransferase
ANOVA: One-way analysis of variance
AST: Aspartate Aminotransferase
*E. kebericho*: *Echinops kebericho*
EO: Essential oil
GHS: Globally Harmonized System of Classification and Labeling of Chemicals
OECD: Organization for Economic Cooperation and Development
ROW: Relative organ’s weight
